# Dynamic kinetics of graft-derived cell-free DNA and personalized tacrolimus target ranges for acute rejection monitoring in pediatric liver transplantation

**DOI:** 10.3389/fphar.2026.1939581

**Published:** 2026-09-30

**Authors:** Meiling Yan, Jia Zhao, Wei Gao, Yinpeng Qin, Jun Lu, Yi Zhang

**Affiliations:** 1 Department of Science and Education, Tianjin First Central Hospital, Tianjin, China; 2 Department of Pharmacy, Tianjin Chest Hospital, Tianjin, China; 3 Department of Liver Transplantation, Organ Transplantation Center, Tianjin First Central Hospital, Tianjin, China; 4 S&KM Biotechnology Co., Ltd., Chengdu, Sichuan, China

**Keywords:** acute rejection, graft-derived cell-free DNA, monitoring, pediatric liver transplantation, tacrolimus trough concentration

## Abstract

**Introduction:**

Acute rejection remains the most common complication during the early post-transplantation period for pediatric patients. Given the limitations of current diagnostic strategies, there is an urgent need to develop accurate and noninvasive biomarkers for allograft monitoring. Tacrolimus-based immunosuppression represents the cornerstone regimen following liver transplantation. However, for pediatric patients, current evidence remains insufficient to establish well-defined therapeutic target ranges for personalized tacrolimus therapy in pediatric patients. We investigated perioperative graft-derived cell-free DNA (GcfDNA) kinetics in pediatric recipients to establish the utility of GcfDNA for monitoring acute rejection and to explore the tacrolimus target concentration range in this population.

**Methods:**

Forty children (age ≤ 3 years) with biliary atresia who underwent liver transplantation were enrolled. All received a tacrolimus-based immunosuppressive regimen on postoperative day (POD) 1 and were followed for at least 1 month. GcfDNA levels were measured by droplet digital PCR.

**Results:**

GcfDNA (%) declined significantly during the first month. Median percentages on POD 1, 7, 14, 21, and 28 were 41.40%, 12.93%, 6.86%, 5.95%, and 3.23%, respectively. The acute rejection group maintained higher GcfDNA levels, with significant differences observed at POD 14, 21, and 28 (*P* < 0.001). GcfDNA (%) was strongly correlated with rejection activity index score (r = 0.854, *P* < 0.001). In this exploratory single-center analysis, receiver operating characteristic (ROC) curve analysis identified an optimal GcfDNA cutoff of 10.21% for predicting acute rejection, with an area under the curve (AUC) of 0.757 (95% CI: 0.691–0.823, *P* < 0.001). The optimal tacrolimus trough concentration (C_0_) threshold was 7.75 ng/mL (AUC = 0.732, 95% CI: 0.655–0.810, *P* < 0.001).

**Conclusion:**

We suggest that GcfDNA may serve as a potential biomarker for acute rejection monitoring in pediatric liver transplantation, with an exploratory predictive cutoff value of 10.21% identified in this single-center cohort. Our findings also tentatively point to a minimal effective tacrolimus C_0_ of 7.75 ng/mL within the first month post-transplantation, alongside an upper safety limit of 10 ng/mL that is primarily grounded in published peer-reviewed literatures. These thresholds, however, were optimized against stable grafts rather than the full spectrum of graft injury and require multicenter validation before clinical adoption.

## 1 Introduction

Liver transplantation is a lifesaving procedure for children with acute or chronic end-stage liver disease, selected liver tumors, and certain metabolic diseases for which transplantation can significantly improve quality of life (Smith and Miloh, 2022). The indications for liver transplantation for children are different than for adults, with biliary atresia the leading cause of liver failure in children, accounting for 50%–70% of patients (Kasahara and Sakamoto, 2025; Capone et al., 2016). Advances in surgical techniques, improved postoperative management, and immunosuppression regimes have led to excellent long-term graft and patient survival, such that over 75%–90% of recipients survive 5 years after transplantation (Wang et al., 2024). However, the success of pediatric liver transplantation does pose the enduring challenge of long-term patient management.

Following liver transplantation, there is a risk of occurrence of transplant-related complications such as rejection and graft function decline that may lead to graft loss and reduced patient survival (Zhou et al., 2022). Thus, monitoring the status of transplanted liver is vital for the long-term survival of recipients. Among these concerns, the expeditious detection of acute rejection is especially critical for long-term outcomes. Conventional liver function tests lack specificity and exhibit a time lag in detecting acute rejection. Although invasive liver biopsy is the diagnostic gold standard for rejection, it carries risks of serious complications (Zhang et al., 2024). Given the limitations of current acute rejection diagnosis, there is a pressing need to develop accurate, noninvasive biomarkers for monitoring allograft quality and function.

The need for life-long immunosuppression and its associated side effects is another major challenge to the long-term survival of pediatric transplantation recipients. Tacrolimus-based immunosuppression, used for over 80% of patients, is the cornerstone regimen following liver transplantation (Kwong et al., 2025). Despite advances in the optimization of immunosuppressive therapies, acute rejection and serious adverse drug reactions to tacrolimus still contribute to morbidity and mortality (Riva et al., 2018). Therapeutic drug monitoring (TDM) for tacrolimus is widely used in clinical practice to personalize therapy by adjusting the dose based on target concentration ranges. However, the latest consensus report on TDM of tacrolimus-personalized therapy indicates that there is still insufficient clinical evidence to establish specific target ranges for pediatric patients (Brunet et al., 2019).

Cell-free DNA is a marker of cell death; therefore, it can be released from necrotic or apoptotic cells in a transplanted organ or from donor-derived hematopoietic cells into the recipient’s blood (Zhong et al., 2025). Recently, graft-derived cell-free DNA (GcfDNA) has emerged as a novel noninvasive biomarker for monitoring rejection and other types of graft injury, offering a new approach for continuous and minimally invasive monitoring (Zhang et al., 2024). GcfDNA levels significantly increase during acute rejection, ischemia-reperfusion injury, and early graft dysfunction after transplantation (Zhong et al., 2025). Acute rejection, including cellular and antibody-mediated rejection, is correlated with a significant elevation of GcfDNA levels in heart, liver, kidney, and lung transplantation (Sebastian et al., 2024). Furthermore, dynamic changes in GcfDNA levels are closely associated with the effectiveness of immunosuppression (Charlton et al., 2018; VanWagner et al., 2016; O'Leary et al., 2016).

To date, most relevant studies of GcfDNA have been conducted in adult liver transplant recipients; however, the kinetic profiles of GcfDNA remain completely uncharacterized in children. Meanwhile, tacrolimus target trough concentrations in pediatric liver transplant recipients remain largely empirical, often extrapolated from adult data rather than derived from pediatric-specific outcomes. No precise concentration ranges have been established by reverse inference using objective rejection biomarkers such as GcfDNA. To address these gaps, we prospectively measured perioperative GcfDNA levels in pediatric liver transplant recipients and evaluated its diagnostic performance for acute rejection. Through this lens, we offer guidance for the use of GcfDNA to monitor acute rejection and potentially define the target concentration range of tacrolimus in the pediatric liver transplant population.

## 2 Methods

### 2.1 Study design

This prospective observational study was conducted at Tianjin First Central Hospital from October 2022 to April 2025. The Ethics Committee of Tianjin First Central Hospital approved the study (Approval Numbers: 2020N179KY and KY20241030-1). The study was conducted in accordance with the 1964 Helsinki Declaration and its later amendments or comparable ethical standards. The guardians of all participants were provided with information about the study, and they provided written informed consent prior to enrollment.

Briefly, for laboratory tests all the pediatric patients undergoing living donor liver transplantation (LDLT) provided blood samples collected on the 1st, 7th, 14th, 21st, and 28th days after surgery. In addition, leftover blood samples were collected after the laboratory tests. For patients diagnosed by liver tissue biopsy as having acute rejection, the pathological results of the liver biopsy were collected. All biopsy samples were assessed in compliance with the Banff classification criteria for hepatic allograft rejection (Demetris et al., 2016). To reduce inter-observer discrepancy, all specimens were independently reviewed by two pathologists in a blinded fashion. The severity of acute rejection was quantified via the Banff Rejection Activity Index (RAI); lesions were further stratified into mild, moderate or severe grades based on the degree of inflammatory infiltration as well as biliary and venous endothelial damage.

### 2.2 Inclusion/exclusion criteria

The main inclusion criteria of all patients undergoing LDLT were the following: age ≤ 3 years, primary disease of biliary atresia, receiving tacrolimus-based immunosuppressive regimens after transplantation, taking tacrolimus from postoperative day 1, and follow-up period ≥ 1 month. Patients were excluded if they: (1) underwent combined organ transplantation; (2) developed complications during follow-up, including drug-induced liver injury, liver abscess, active biliary complications or abdominal infection; (3) received drugs that significantly affect tacrolimus blood concentration, including cytochrome P450 (CYP) 3 A inducers (phenobarbital, carbamazepine) and CYP3A inhibitors (voriconazole, posaconazole); (4) had a follow-up duration of less than 1 month; (5) or switched to other immunosuppressants during follow-up. Samples with potential pre-analytical contamination, hemolysis, or insufficient plasma quality were not used for GcfDNA testing, and the sample processing workflow was standardized to reduce leukocyte lysis and cfDNA contamination.

### 2.3 Data items

We collected data on the patient’s sex, age, height, weight, primary disease, surgical approach, and weight of the transplanted liver. Clinical laboratory assessment included routine blood tests, liver function tests, and tacrolimus trough concentrations, all of which were measured on the 1st, 7th, 14th, 21st, and 28th day after the operation.

### 2.4 cfDNA extraction and measurement

Circulating cfDNA was isolated from 2 mL of plasma using the NucleoSnap DNA Plasma kit (Macherey-Nagel, Düren, Germany) according to the manufacturer’s instructions. The cfDNA was eluted from spin columns in 40 μL nuclease-free water. The fractional abundance of cfDNA was measured by a technical testing service (S&KM Biotechnology Co., Ltd., Chengdu, China [www.skmbio.com]) (Di et al., 2021). In brief, SNP assays were used to identify a set of 16 preselected SNPs which have a known and validated minor allele frequency of >45% in Chinese populations; the SNPs would be used to differentiate between donor and graft DNA isolated from plasma samples.

For each droplet digital PCR (ddPCR) assay, 3 μL cfDNA sample, 1 μL primer/probe mix, 10 μL ddPCR supermix for probes (Bio-Rad, Hercules, CA, USA), and 6 μL RNase-free water were added to a 20-μL reaction mixture. Droplets were then generated by a QX200 droplet generator device (Bio-Rad) using 20 μL of mixture and 70 μL of droplet generation oil for probes. The cycling conditions were as follows: 10 min at 95 °C, 40 cycles of denaturation at 94 °C for 30 s, annealing/extension at 60 °C for 1 min, and a final step at 98 °C for 10 min. Droplets were then read in the droplet reader and analyzed using QuantaSoft™ software (Bio-Rad). Individualized ddPCR tests were used to monitor GcfDNA percent over time for each patient.

### 2.5 Genotype analyses

DNA was extracted from peripheral blood, and the donors’ and recipients’ CYP3A5 genotypes were determined by PCR. The primers for CYP3A5*3 (rs776746) were 5′- GAAGCAAGTGGGAGAA AG-3′(forward primer) and 5′- TGA​TGA​AGG​GTA​ATG​TGG-3′ (reverse primer).

### 2.6 Statistical analysis

GcfDNA fractions (%) were calculated using the following formula:

$$\text{GcfDNA }\left(\%\right)=\frac{\begin{array}{c} \text{Donor}\;\text{cfDNA}\;(\text{copy}\;\text{number}\;\text{of}\;\text{homozygous}\;\text{genotype}\\ \text{representing}\;\text{donor}\;\text{derived}\;\text{cfDNA}\;\text{in}\;\text{selected}\;\text{SNP}) \end{array}}{\text{Total}\;\text{cfDNA}\;\text{total}\;\text{copy}\;\text{number}\;\text{of}\;\text{selected}\;\text{SNP}\;}$$

Statistical analyses were conducted with SPSS software version 26.0 (IBM Corp., Armonk, NY), and all graphs were created using GraphPad Prism 10.0 software (GraphPad Software, Inc., San Diego, CA, USA). Continuous data that followed a normal distribution are presented as mean ± standard deviation, whereas data that did not follow a normal distribution are expressed as median (interquartile range: Q1, Q3). The Mann-Whitney *U*-test was used to compare GcfDNA fraction (%) between different groups. Comparisons between groups for normally distributed quantitative data were performed using independent samples *t* tests, whereas the Mann-Whitney *U-*test was used for non-normally distributed data. Categorical data were summarized as frequency (percent), and group comparisons were conducted using χ^2^ tests or Fisher’s exact test, as appropriate. Receiver operating characteristics (ROC) curves were computed to assess the diagnostic performance of the analyzed biomarkers, and optimal cutoff points were calculated using the Youden method. To account for within-patient clustering of repeated GcfDNA measurements, generalized estimating equations (GEE) with a logistic link and robust sandwich standard errors were used to estimate the population-averaged effect of GcfDNA on acute rejection. For ROC analysis, a patient-level cluster bootstrap (2,000 iterations, patient-level resampling with replacement) was employed to derive statistically valid 95% confidence intervals for the AUC, sensitivity, and specificity. Because of the absence of an independent holdout validation cohort, we implemented leave-one-out cross-validation (LOOCV) to internally validate the established ROC-derived predictive model. Longitudinal changes in GcfDNA levels across postoperative days 1, 7, 14, 21, and 28 were analyzed using the Friedman test, as the data were paired repeated measurements and did not meet the assumptions required for repeated-measures analysis of variance (ANOVA). When the Friedman test showed an overall significant difference, post-hoc pairwise comparisons were performed using Bonferroni-adjusted pairwise comparisons. A *P* < 0.05 was considered statistically significant.

## 3 Results

### 3.1 Patient demographics and baseline characteristics

We screened 65 patients. Excluded patients included four who were over 3 years old, eight who had abdominal infection, six patients who had drug-induced liver injury, five patients who changed tacrolimus-based immunosuppressive regimens, and two patients who had incomplete clinical data. Ultimately, 40 patients were included in the study. According to whether rejection occurred during the follow-up period, we divided the patients into an acute rejection group (n = 20) and a nonacute rejection group (n = 20). The study-screening process and the reasons for exclusion are presented in Supplementary Figure 1. Table 1 presents patient demographic and baseline characteristics. There were no statistically significant differences in baseline characteristics, including sex, age, weight, and body mass index (BMI), between the two groups (all *P* > 0.05).

**TABLE 1:** Basic information for acute and nonacute rejection groups (n = 40).

Characteristics	Acute rejection group (n = 20)	Nonacute rejection group (n = 20)	*P* Value
Sex (male/%)	9 (39.1%)	8 (47.1%)	0.749
Age (years)	0.6 (0.5, 0.8)	0.6 (0.5, 1.0)	0.526
Weight (kg)	7.0 (6.0, 7.5)	6.5 (6.0, 9.7)	0.794
BMI (kg/m^2^)	15.3 (15.0, 16.1)	15.6 (15.3, 16.5)	0.165

### 3.2 Elimination kinetics of GcfDNA in pediatric patients after LDLT

To characterize the physiological elimination kinetics of GcfDNA following pediatric LDLT, we performed serial measurements of GcfDNA percent in a cohort of 20 transplant recipients who did not experience acute rejection, at postoperative day (POD) 1, 7, 14, 21, and 28. In this cohort, GcfDNA (%) demonstrated a significant declining trend, with plasma elimination kinetics characterized by an “L-shaped” curve. The median percent of GcfDNA on POD 1, 7, 14, 21, and 28 was 41.40% (IQR, 19.77%–49.94%), 12.93% (IQR, 7.79%–16.49%), 6.86% (IQR, 4.54%–10.82%), 5.95% (IQR, 2.90%–7.53%), and 3.23% (IQR, 2.25%–4.41%), respectively (Figure 1). The Friedman test demonstrated a significant overall difference in GcfDNA levels across postoperative time points. Post-hoc Bonferroni-adjusted pairwise comparisons showed that GcfDNA levels at POD28 were significantly lower than those at POD14, POD7, and POD1. However, we did not observe a significant difference between POD21 and POD28 after Bonferroni correction (adjusted *P* = 1.000). These findings suggest that GcfDNA decreased after surgery and appeared to reach a relatively stable plateau during the later postoperative period.

**FIGURE 1:**
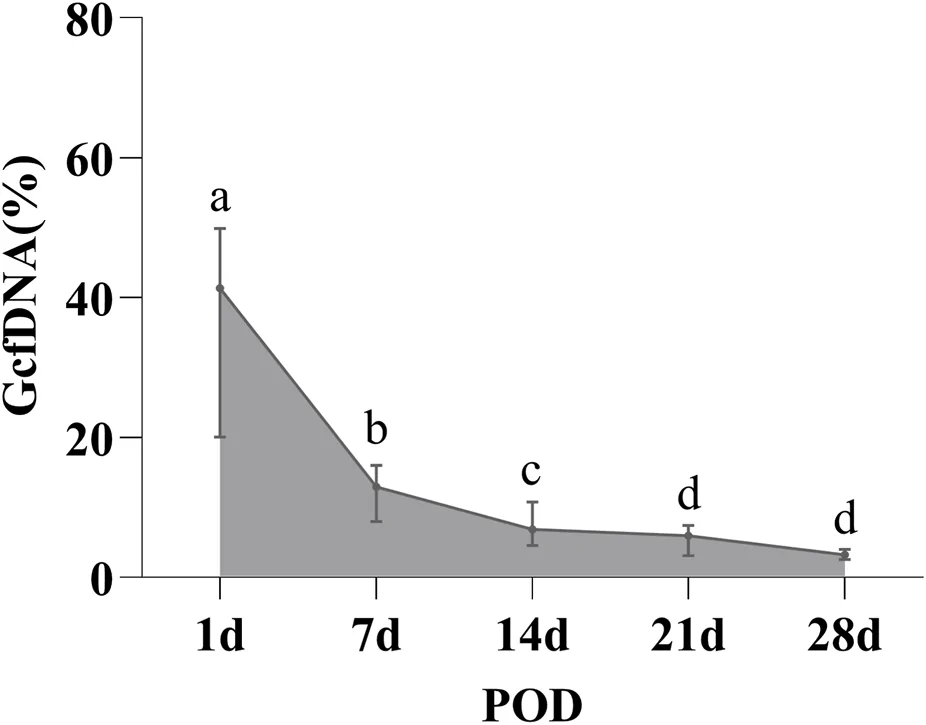
Elimination kinetics of GcfDNA in nonacute rejection patients after transplantation. Data are represented as GcfDNA (%). The Friedman test was applied to evaluate overall differences in GcfDNA (%) across postoperative time points, followed by post-hoc pairwise comparisons with Bonferroni correction for multiple testing. Different letters above the data points indicate statistically significant differences between postoperative time points (Bonferroni-corrected *P* < 0.05); shared letters indicate no significant difference. Abbreviations: GcfDNA, graft-derived cell-free DNA; POD, postoperative day.

### 3.3 The GcfDNA monitoring in pediatric patients after LDLT

During 1 month after LDLT, we performed 212 GcfDNA assays, with 112 assays derived from the acute rejection group and 100 from the nonacute rejection group. The GcfDNA results across all assays ranged from 0.21% to 97.48%. The median GcfDNA percent was 19.21% (IQR, 11.08%–38.41%) for acute rejection and 7.63% (IQR, 3.95%–15.04%) for nonacute rejection, with a significantly higher GcfDNA percent in the acute rejection group (*P* < 0.001; Figure 2A). Comparison of GcfDNA (%) between the acute and nonacute rejection groups at POD 1, 7, 14, 21, and 28 demonstrated that the acute rejection group maintained consistently higher levels throughout the observation period. Notably, significant inter-group differences were specifically identified at POD 14, 21, and 28 (*P* < 0.001; Figure 2B). Individual longitudinal trajectories of GcfDNA (%) from POD 1 through POD 28 for all 20 recipients with biopsy-proven acute rejection are presented in Supplementary Figure 2.

**FIGURE 2:**
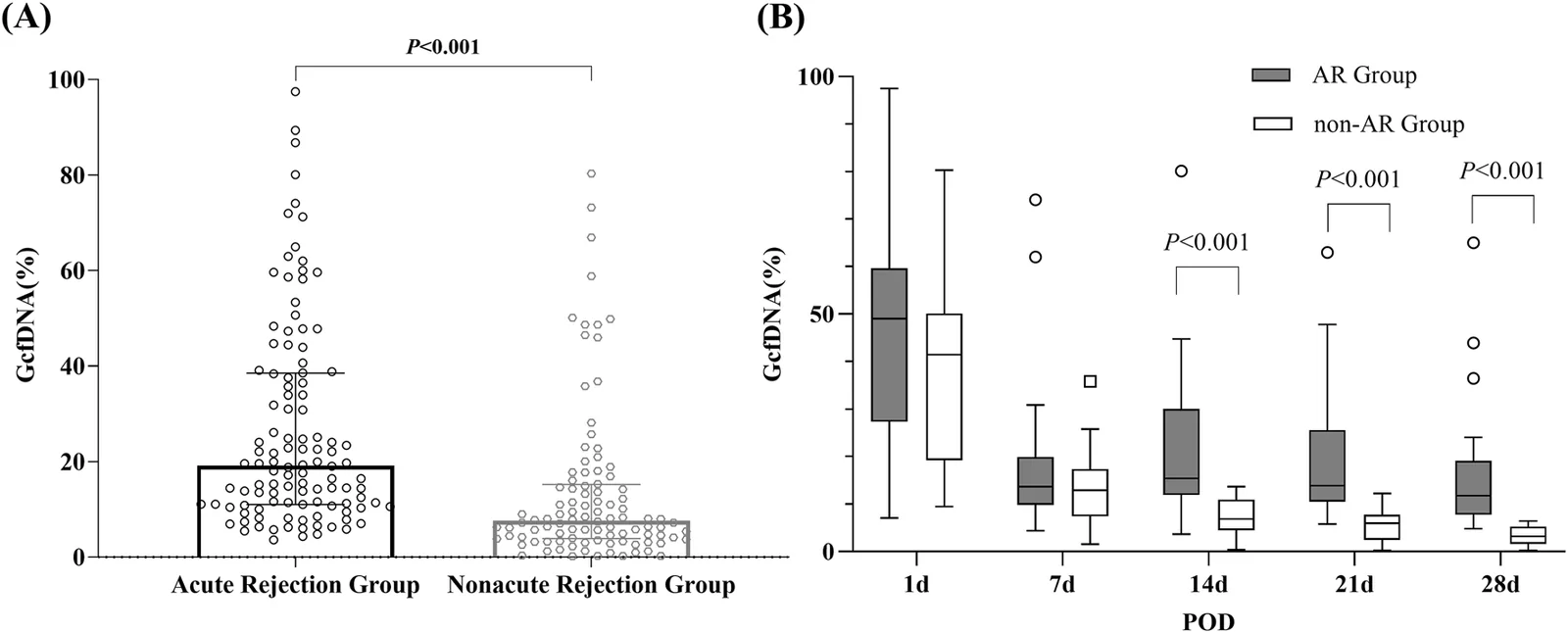
GcfDNA levels in acute and nonacute rejection groups at 1 month post-transplantation **(A)** GcfDNA (%) difference between acute rejection group and nonacute rejection group, All recipients underwent protocol-scheduled GcfDNA testing at five postoperative time points (POD 1, 7, 14, 21, and 28). A total of 100 measurements from 20 non-rejection recipients (one observation per patient per time point) were included. For the 20 patients with biopsy-proven acute rejection, 112 measurements were analyzed, comprising 100 scheduled samples and 12 additional samples obtained at the time of clinically indicated liver biopsy **(B)** Box and whisker plot analysis of differences in GcfDNA (%) on POD1, 7, 14, 21, 28 between acute rejection group and nonacute rejection group. Box-and-whisker plots were constructed using the Tukey method, with whiskers extending to the farthest data points within 1.5×IQR; outliers beyond this range were plotted as individual symbols. Between-group differences in GcfDNA (%) were compared using the Mann-Whitney *U* test. Abbreviations: GcfDNA, graft-derived cell-free DNA; POD, postoperative day.

### 3.4 Correlation between GcfDNA and the severity of acute rejection

To analyze the association between GcfDNA and the severity of acute rejection, we performed statistical analysis using GcfDNA (%) measured on the day of liver biopsy. Detailed clinical and pathological characteristics of all 20 biopsy-proven acute rejection episodes are summarized in Supplementary Table 1. We found a strong correlation between GcfDNA (%) and the RAI score for patients with acute rejection (r = 0.854, *P* < 0.001; Figure 3A). Higher grades of acute rejection were associated with elevated GcfDNA (%). The median GcfDNA (%) for borderline, mild, moderate, and severe rejection was 20.35% (IQR, 16.45%–23.16%), 34.71% (IQR, 24.54%–38.31%), 46.16% (IQR, 37.76%–62.61%), and 63.98% (IQR, 47.05%–82.44%), respectively. The GcfDNA (%) in the severe acute rejection group was significantly different compared with the other three groups (*P* = 0.001 vs. borderline acute rejection, *P* = 0.018 vs. mild acute rejection, *P* = 0.01 vs. moderate acute rejection; Figure 3B).

**FIGURE 3:**
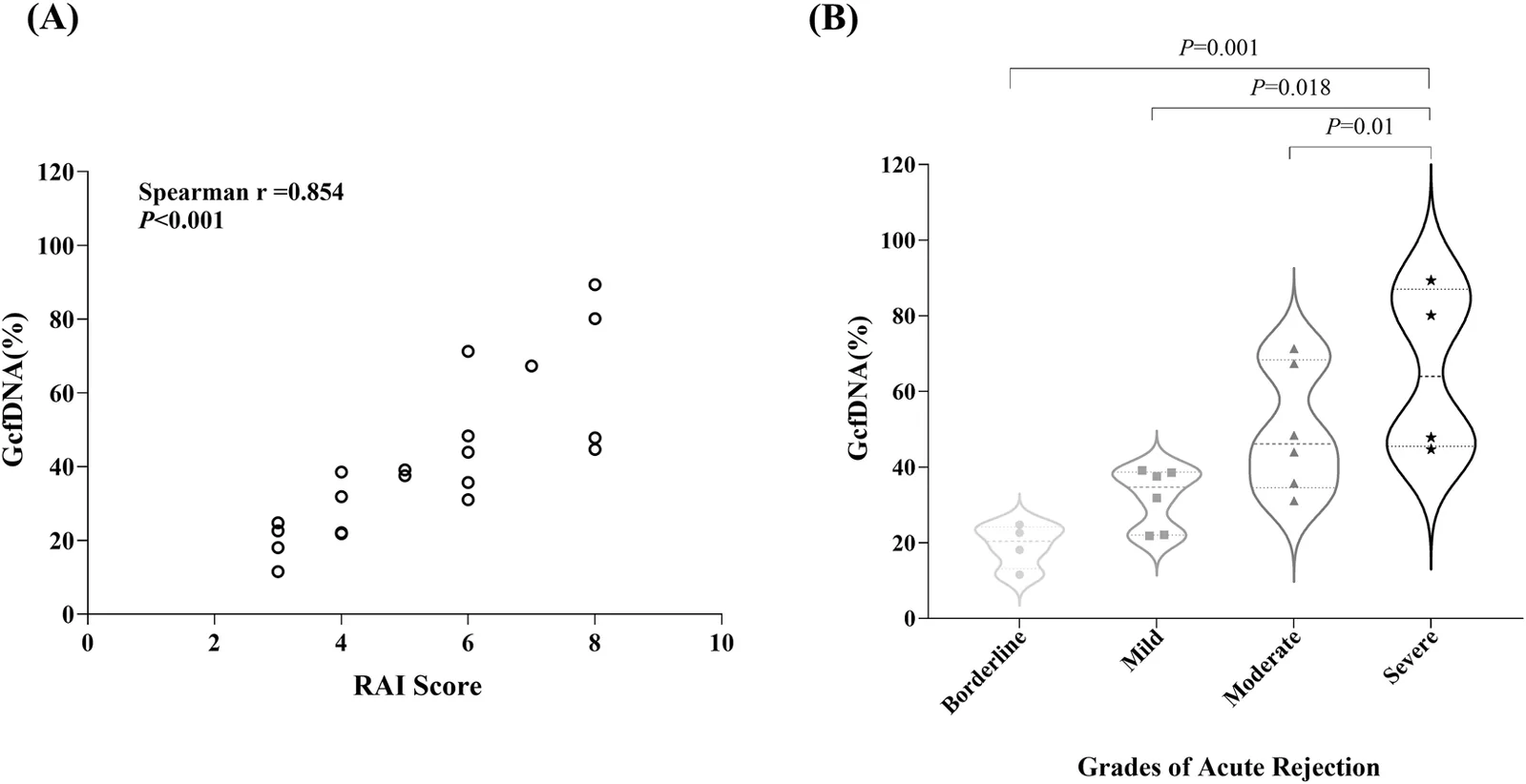
Relation between GcfDNA level and severity of rejection **(A)** Correlation analysis between GcfDNA (%) and RAI Score **(B)** The relationship between GcfDNA (%) with severity of acute rejection. Spearman rank correlation analysis was conducted to assess the association between peribiopsy GcfDNA (%) and RAI score. Abbreviations: GcfDNA, graft-derived cell-free DNA; RAI, rejection activity index.

### 3.5 GcfDNA for predicting acute rejection after pediatric liver transplantation

The cut-off value of GcfDNA (%) for predicting acute rejection was determined using a ROC curve; these diagnostic thresholds were derived from a single-center cohort and optimized against stable graft status. The optimal cut-off was defined as the point that maximized the Youden index (Youden index = 0.43), which corresponded to 10.21% (areas under the ROC curves [AUC] = 0.757, 95% CI: 0.691–0.823, *P* < 0.001, n = 40), yielding a sensitivity of 0.804, a specificity of 0.630, a positive predictive value (PPV) of 0.709 and a negative predictive value (NPV) of 0.741 (Figure 4A). GEE with a logistic link and robust sandwich standard errors showed that GcfDNA (%) was a significant population-averaged predictor of acute rejection (OR = 1.041 per 1% increase, 95% CI: 1.021–1.061, *P* < 0.0001). Patient-level cluster bootstrap ROC analysis yielded an AUC of 0.757 (95% CI: 0.699–0.816), with a corresponding sensitivity of 0.804 (95% CI: 0.732–0.870) and specificity of 0.630 (95% CI: 0.537–0.717). For the patient-level sensitivity analysis, we used the first scheduled GcfDNA measurement obtained on POD 14, such that each recipient contributed a single independent observation. The corresponding patient-level ROC analysis yielded an AUC of 0.887 (95% CI: 0.760–0.982). A total of 212 assays were included in the LOOCV ROC analysis, including 112 assays derived from the acute rejection group and 100 from the nonacute rejection group. The LOOCV-validated AUC was 0.739 (95% CI, 0.668–0.802). The optimal cutoff of the LOOCV-predicted probability was 0.439, with a Youden index of 0.424. At this cutoff, the sensitivity was 0.804, specificity was 0.620, accuracy was 0.717, PPV was 0.703, and NPV was 0.738. To compare the predictive performance of GcfDNA (%) with standard liver function tests for acute rejection, we generated ROC curves for alanine aminotransferase (ALT), aspartate aminotransferase (AST), gamma-glutamyl transpeptidase (GGT), alkaline phosphatase (ALP), and total bilirubin (TBIL). The AUCs were 0.699 (95% CI: 0.628–0.770, *P* < 0.001, n = 40) for ALT, 0.649 (95% CI: 0.575–0.723, *P* < 0.001, n = 40) for AST, 0.737 (95% CI: 0.670–0.803, *P* < 0.001, n = 40) for GGT, 0.617 (95% CI: 0.542–0.692, *P* = 0.003, n = 40) for ALP, and 0.585 (95% CI: 0.508–0.663, *P* = 0.032, n = 40) for TBIL. Comparative analysis revealed that GcfDNA (%) exhibited a higher AUC than all individual liver function markers, demonstrating its superior ability to predict acute rejection (Figure 4B).

**FIGURE 4:**
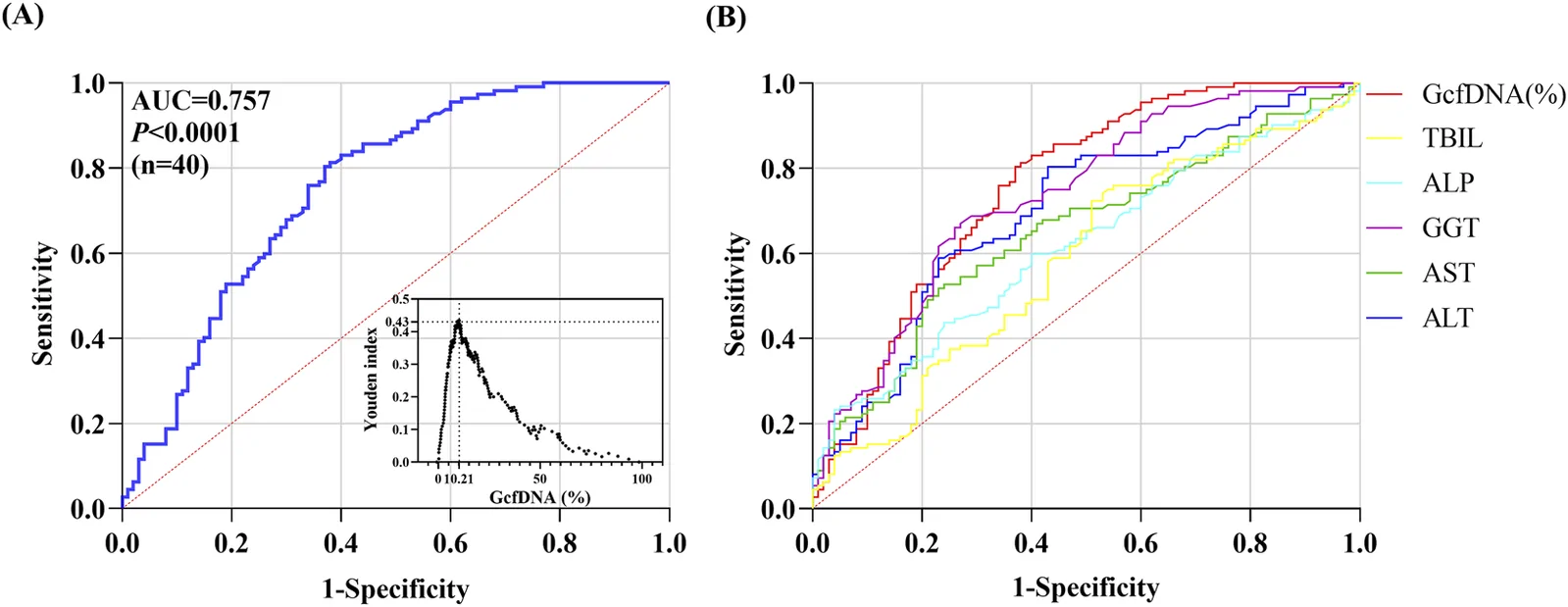
ROC curves of GcfDNA percent and liver function tests for acute rejection prevention **(A)** ROC curve of GcfDNA (%) **(B)** ROC curve of liver function tests. ROC analysis was conducted using 212 serial samples obtained from 40 patients. ROC curve analysis was performed to determine the optimal GcfDNA cutoff for predicting acute rejection, with the threshold defined by maximizing the Youden index. Abbreviations: ALP, alkaline phosphatase; ALT, alanine aminotransferase; AST, aspartate aminotransferase; AUC, area under the curve; GcfDNA, graft-derived cell-free DNA; GGT, gamma-glutamyl transpeptidase; ROC, receiver operating characteristics; TBIL, total bilirubin.

### 3.6 GcfDNA for reexamining effective tacrolimus trough concentrations

Tacrolimus trough concentrations (Tac C_0_) were maintained above the lower limit of the therapeutic range (7 ng/mL) used by our institution’s transplant surgeons to guide dose adjustments during the first 4 weeks following LDLT. On the basis of the established cut-off value of 10.21% for GcfDNA (%) and the lower limit of 7 ng/mL for the Tac target concentration, we divided the patients into four groups: (1) Tac C_0_ < 7 ng/mL and GcfDNA (%) <10.21%, (2) Tac C_0_ < 7 ng/mL and GcfDNA (%) ≥10.21%, (3) Tac C_0_ ≥ 7 ng/mL and GcfDNA (%) <10.21%, and (4) Tac C_0_ ≥ 7 ng/mL and GcfDNA (%) ≥10.21%. The number of sample episodes in each group was counted, and Fisher’s exact test was performed. The proportion of samples in the group with Tac C_0_ < 7 ng/mL and GcfDNA (%) ≥10.21% was significantly higher than that in the group with Tac C_0_ < 7 ng/mL and GcfDNA (%) <10.21% (72.37% vs. 27.63%). Similarly, the proportion of samples in the group with Tac C_0_ ≥ 7 ng/mL and GcfDNA (%) <10.21% was significantly higher than that in the group with Tac C_0_ ≥ 7 ng/mL and GcfDNA (%) ≥10.21% (70.83% vs. 29.17%) (Fisher’s *P* < 0.001; Figure 5A). The Tac C_0_ was determined using ROC curve analysis, yielding an optimal threshold of 7.75 ng/mL at the maximum Youden index (Youden index = 0.44) (AUC = 0.732, 95% CI: 0.655–0.810, *P* < 0.001), with a sensitivity of 0.687 and a specificity of 0.750 (Figure 5B).

**FIGURE 5:**
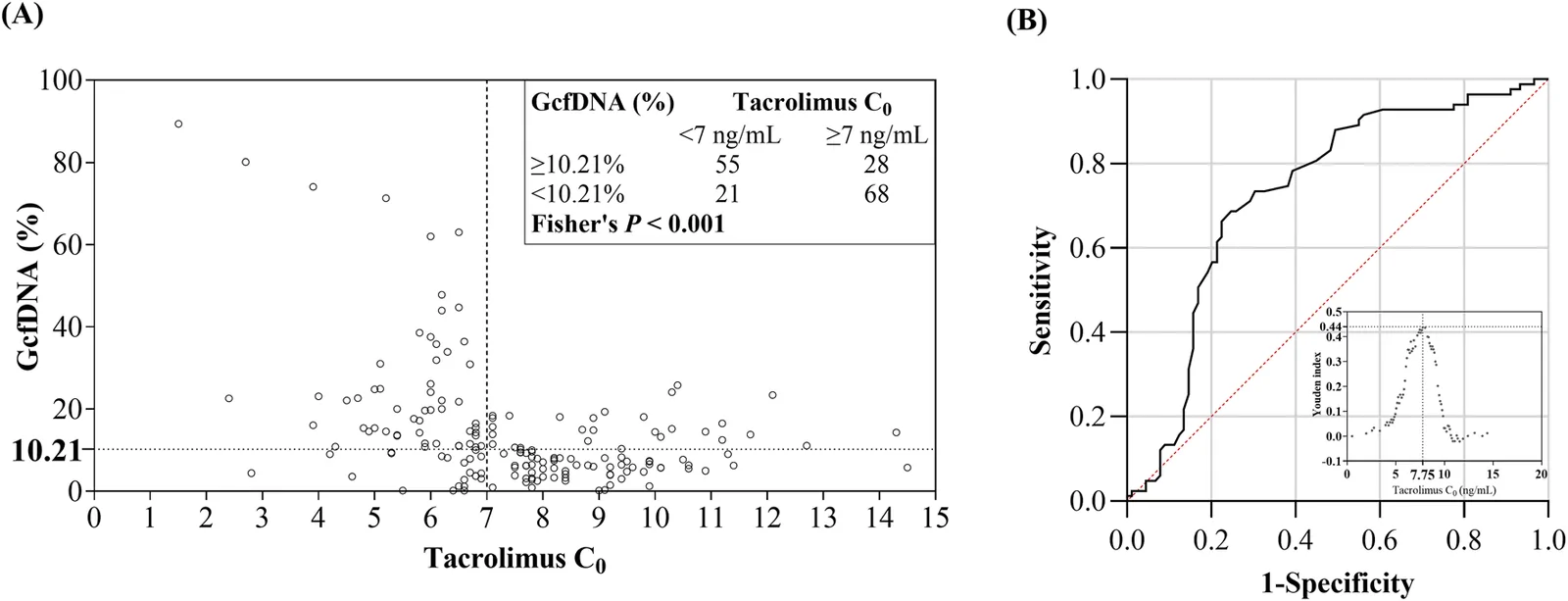
ROC curve of GcfDNA for determining the lowest effective tacrolimus concentration **(A)** Effect relation between GcfDNA and tacrolimus C_0_ **(B)** ROC curve of GcfDNA (%) for predicting tacrolimus C_0_ (ng/mL). ROC analysis was conducted using 212 serial samples obtained from 40 patients. Fisher’s exact test was applied to compare the proportional distribution of samples across the four groups. ROC curve analysis was conducted to derive the optimal tacrolimus C_0_ threshold for predicting acute rejection, with the cutoff value defined by maximizing the Youden index. Abbreviations: C_0_, trough concentrations; GcfDNA, graft-derived cell-free DNA; ROC, receiver operating characteristics.

### 3.7 Associations of CYP3A5*3 polymorphisms with tacrolimus exposure and GcfDNA

We performed CYP3A5*3 genotyping of 40 pediatric LDLT donor-recipient pairs. The wild-type homozygous genotype at the *CYP3A5* -6986 A>G locus was defined as AA (*1*1), the heterozygous variant as AG (*1*3), and the homozygous mutant as GG (*3*3). Four recipients carried CYP3A5*1*1, 15 carried CYP3A5*1*3, and 21 carried CYP3A5*3*3. Among donors, six were CYP3A5*1*1, 17 were CYP3A5*1*3, and another 17 were CYP3A5*3*3 (Supplementary Figure 3). The allelic frequency of the G variant was 67.5%. Using χ^2^ goodness-of-fit test, we found that genotype distributions for both donors and recipients conformed to the Hardy–Weinberg equilibrium (*P* > 0.05).

We stratified recipients into the *CYP3A5*-expressing group (CYP3A5*1/*1 and CYP3A5*1/*3, n = 19) and non-expressing group (CYP3A5*3/*3, n = 21) according to the presence or absence of the CYP3A5*1 allele. Stratified by either recipient or donor *CYP3A5* genotype, we found that significantly higher tacrolimus trough concentration/daily dose (C_0_/D) ratios were consistently identified in the *CYP3A5* nonexpressing group relative to the *CYP3A5*-expressing group at all predefined postoperative time points (all *P* < 0.001). When stratified by recipient *CYP3A5* genotype, we found that the percent of GcfDNA was significantly lower in the *CYP3A5*-nonexpressing group versus the *CYP3A5*-expressing group at POD 7 and POD 14 (*P* < 0.05). Stratification according to donor *CYP3A5* genotype revealed significantly reduced GcfDNA (%) in the nonexpressing cohort only at POD 7 (*P* < 0.05).

## 4 Discussion

To the best of our knowledge, this study is the first based on GcfDNA for acute rejection monitoring and reevaluating clinically effective thresholds for tacrolimus trough concentrations for pediatric liver transplantation. Our findings demonstrate the feasibility of the simplified GcfDNA method as a noninvasive, practical, rapid, and relatively inexpensive tool for monitoring acute rejection in children. Furthermore, these results support exploratory evaluation of the target trough concentrations of tacrolimus, which are essential for maintaining graft function in pediatric liver transplant recipients.

Living donor liver transplantation (LDLT) is commonly performed today for pediatric patients with biliary atresia, and it has become the gold standard for treating pediatric patients who have end-stage liver disease (Meng et al., 2019). The latest survey showed 1-, 3-, and 5-year graft survival rates among pediatric LDLT recipients were 94.3%, 92.4%, and 90.3%, respectively (Kwong et al., 2025). However, graft injuries, including ischemia-reperfusion, infection, or acute rejection, have direct effects on patient survival following LDLT. Acute rejection is the most common complication in the first year, affecting up to 40% of recipients (Kanneganti et al., 2022). Rejection occurs most frequently within 7–14 days following liver transplantation (Bonaccorsi-Riani et al., 2016). Timely detection of acute rejection is crucial for ensuring optimal long-term outcomes. Although conventional liver function tests (LFTs) are useful for monitoring graft function, they lack the specificity to diagnose either antibody-mediated rejection or acute cellular rejection following transplantation, and LFTs have a significant time lag. Consequently, liver biopsy is often required for a definitive diagnosis (Neil and Hübscher, 2010). However, biopsy itself is an invasive procedure associated with risks of pain, bleeding, sepsis, and biliary fistula, which can threaten the recipient’s quality of life. Apart from the potential risks and inconvenience, repeated serial biopsies are costly, prone to sampling error, and have limited specificity and sensitivity because of subjective interpretation (Tang et al., 2017). Consequently, it is imperative to develop novel, noninvasive biomarkers with superior accuracy for monitoring allograft status in pediatric liver transplant recipients.

In the present study, on POD 1 following transplantation, GcfDNA rapidly increased to 41.40% (19.77%–49.94%) as a result of ischemia-reperfusion injury. Subsequently, as graft function gradually recovered, the GcfDNA levels in patients who did not experience acute rejection declined to 12.93% (7.79%–16.49%) by 1 week after transplantation. Schütz et al. reported that the GcfDNA percentages were highly elevated on POD 1, most likely reflecting ischemia-reperfusion damage, but the median GcfDNA percentages decreased within the first 7–10 days to a level below 10% in stable patients with no signs of graft injury (Schütz et al., 2017). Our results concerning GcfDNA changes agree with the previous study (Macher et al., 2016; Beck et al., 2013). We observed significant differences in GcfDNA level between the acute rejection and nonacute rejection groups, specifically identified at POD 14, 21, and 28. Consistent with previous findings, the first week after liver transplantation is characterized by high baseline GcfDNA release related to ischemia-reperfusion injury, graft manipulation, and early graft recovery. In the absence of postoperative complications, the elevated levels of GcfDNA rapidly return to baseline within a few days (Zhong et al., 2025). At POD 7, the acute rejection group showed a numerically higher GcfDNA level, but the difference did not reach statistical significance; thus, the biomarker was not reliable enough for stand-alone discrimination at that time point in this cohort. Accordingly, GcfDNA measured within the first 7 days post-transplantation has limited utility for the early detection of acute rejection. For measurements obtained on POD 1 to 7, the primary clinical utility of GcfDNA in our cohort was in establishing individual baseline trajectories and flagging aberrations from the expected postoperative downward trend, instead of defining a definitive diagnostic threshold for acute rejection. Studies indicate that reflecting the severity of rejection, GcfDNA levels post-liver transplantation are significantly positively correlated with severity of acute rejection (Cox et al., 2022; Sorbini et al., 2025). Our results also showed a strong correlation between GcfDNA (%) and the RAI score of patients who experienced acute rejection (r = 0.854, *P* < 0.001).

The area under the ROC curves yielded a maximum (0.757; 95% CI, 0.691–0.823, *P* < 0.001) at a segregating value of 10.21% for GcfDNA. This analysis supports the decision to use 10.21% GcfDNA as the cut-off value for predicting acute rejection after the initial high-variability period, especially from POD 14 onward. Additionally, compared with conventional LFTs, GcfDNA had higher diagnostic efficacy for acute rejection. Published studies have likewise verified that GcfDNA affords superior and earlier discrimination for acute rejection relative to conventional LFTs for liver transplant recipients, enabling rejection detection as early as 8–15 days before biopsy-confirmed rejection (Schütz et al., 2017). The GcfDNA threshold for predicting acute rejection in adult liver transplant recipients is commonly reported to be within the 10%–20% range, a range that aligns with our findings (Schütz et al., 2017; Fernández-Galán et al., 2022; Levitsky et al., 2022; Oellerich et al., 2014). Deng et al. recently reported an adult liver transplantation study that established a GcfDNA cutoff value of 10.39% for identifying acute rejection, a value highly consistent with our findings (Deng et al., 2026). In contrast, Zhao et al. revealed a discordant GcfDNA threshold of 28.7% for pediatric liver transplant rejection, although this finding was derived from a relatively small number of patients (Zhao et al., 2021). We established the 10.21% cutoff value using a rigorously selected cohort with major non-rejection-related allograft injuries excluded. Although our study design facilitates isolation of signals specifically attributable to acute rejection, it constrains the real-world diagnostic specificity of the aforementioned threshold. We completely acknowledge that in real-world clinical practice, severe infections (such as cytomegalovirus or Epstein-Barr virus reactivation) and biliary complications (such as biliary stricture or leakage) induce graft cell damage and non-specifically elevate GcfDNA levels (Zhong et al., 2025). Zhang et al. demonstrated that, although both rejection and non-rejection conditions elevate GcfDNA levels, non-rejection complications typically exhibit slower, lower-magnitude increases with shorter duration compared with acute rejection (Zhang et al., 2026). For discrimination between acute rejection and allograft dysfunction not related to rejection (ADNR, including biliary complications and other nonrejection insults), available evidence indicates a substantially higher diagnostic cutoff is required. Specifically, the optimal threshold for differentiating acute rejection from ADNR was 20.4%, whereas cutoffs to distinguish acute rejection from stable allograft function generally approximate 10% or lower (Millán et al., 2025). Consistent with recent literature, GcfDNA levels (%) were significantly higher in acute rejection patients versus ADNR and stable graft recipients, and no statistically significant discrepancy existed between the ADNR and stable cohorts. The optimal GcfDNA cutoff of 15.58% was calculated for discrimination of acute rejection from ADNR (Deng et al., 2026).

By excluding confounders, we demonstrate the maximum potential specificity of GcfDNA for acute rejection and suggest that when non-rejection causes of cell death are ruled out, elevated GcfDNA is highly predictive of acute rejection. Therefore, in a complex clinical setting, an isolated elevation in GcfDNA cannot automatically be attributed to rejection without further investigation. Instead of positioning GcfDNA as a standalone definitive diagnostic test that replaces biopsy for all comers, we propose the function of GcfDNA in real-world practice as a high-sensitivity triage tool and dynamic monitor: (1) Even in complex settings, a persistently low level of GcfDNA strongly suggests the absence of significant graft injury (whether from rejection or other causes), which can provide clinicians with confidence to avoid unnecessary biopsies when LFTs are mildly elevated because of known non-rejection factors, e.g., stable biliary stricture or controlled viral load. (2) When GcfDNA is significantly elevated in a patient who has potential confounders, the elevation serves as a “red flag” indicating active, substantial graft damage. This flag should trigger a structured differential diagnosis workflow, e.g., viral PCR, doppler ultrasound, magnetic resonance cholangiopancreatography, instead of immediate empiric anti-rejection therapy. (3) We stress that longitudinal kinetics of GcfDNA provide superior diagnostic value compared with isolated single-point measurements for differentiating rejection from ADNR in complicated clinical scenarios.

Furthermore, using the GcfDNA (%) as a measure of graft integrity, we calculated a limit of 7.75 ng/mL for the lower tacrolimus trough concentration during the first month following LDLT, a value well in line with observations of tacrolimus trough levels from large pediatric cohorts (Ling et al., 2025; Riva et al., 2017). Moreover, this finding is consistent with the established Chinese clinical practice guidelines for pediatric liver transplantation (2026 edition), which recommend maintaining a tacrolimus trough concentration of 8–12 ng/mL 1 month post-transplantation (Chinese Society of Organ Transplantation, Chinese Medical Association, 2026). The 2012 Practice Guidance jointly issued by the American Association for the Study of Liver Diseases and the American Society of Transplantation (AASLD/AST) recommends a tacrolimus target levels of 5–10 ng/mL beyond 3 months after liver transplantation (Lucey et al., 2013). The latest clinical practice guidelines on liver transplantation issued by the European Association for the Study of the Liver (EASL) recommend maintaining tacrolimus trough levels at 6–10 ng/mL within the first month following liver transplantation, with no separate targeted recommendations for pediatric populations (European Association for the Study of the Liver, 2024). In our institution, a slightly lower therapeutic range of 7–10 ng/mL is used to guide dose adjustments during the first month after LDLT. In contrast, many centers target a range of 10–12 ng/mL in the early post-transplant period (Prytula et al., 2016). Given the high prevalence of masked hypertension, systolic and diastolic hypertension are associated with tacrolimus C_0_; thus, tacrolimus C_0_ should be maintained below 10 ng/mL (Prytula et al., 2016). Riva et al. reported that for the most frequent adverse events, i.e., nephrotoxicity and hypomagnesemia, the mean tacrolimus C_0_ was 4.0–9.0 ng/mL, indicating a low risk of exposure-related toxicity (Riva et al., 2017).

For tacrolimus trough concentrations, three reference thresholds are contextualized as follows. The institutional target range of 7–10 ng/mL represents the conventional therapeutic window for pediatric liver transplantation at our center, derived empirically from historical clinical practice and local institutional protocols. The ROC-identified lower threshold of 7.75 ng/mL serves as a data-driven cutoff, below which the risk of biopsy-proven acute rejection rises significantly in our cohort. This value is marginally higher than the institutional lower bound of 7 ng/mL, suggesting that the conventional lower limit may be insufficiently stringent for a subset of patients. Finally, the upper limit of 10 ng/mL aligns with the upper therapeutic boundary recommended in recent peer-reviewed literature. Owing to the limited follow-up duration of this cohort, safety-related adverse event outcomes were not systematically captured. Accordingly, the proposed upper safety threshold remains grounded in published literature rather than being empirically derived from the present study dataset. Therefore, tacrolimus dosing adheres to stratified protocols from transplant institutions and consensus guidelines, with strict compliance with labeling requirements for its narrow therapeutic index, routine trough concentration monitoring and individualized dose adjustment.

Tacrolimus is predominantly metabolized in the liver and small intestine via CYP 3 A isoforms, whereas its transmembrane efflux is mediated by P-glycoprotein (Buendia et al., 2014). For liver transplant populations, it is clinically imperative to independently dissect the respective influences of donor and recipient *CYP3A5* genotypes on tacrolimus pharmacokinetic disposition is clinically imperative. Recipients carrying at least one functional CYP3A5*1 allele (expressers) generally require a 1.5–2 fold higher initial tacrolimus dose relative to homozygous CYP3A5*3/*3 nonexpressers to attain therapeutic target trough concentrations (Khamlek et al., 2024). This information is crucial for interpreting the proposed 7.75 ng/mL lower limit, because expressers may naturally trend toward subtherapeutic levels with standard dosing, thereby increasing the risk of underexposure and potential treatment failure. Although this cutoff value yielded favorable specificity within our study cohort, individualized clinical dosing adjustments should be guided by recipients’ CYP3A5 genotype status. For CYP3A5-expressing patients carrying functional *1 alleles, clinical management prioritizes sufficient tacrolimus exposure to attain or surpass the predefined lower therapeutic threshold. By contrast, CYP3A5 nonexpressors require proactive dose moderation to prevent excessive drug accumulation and associated toxicities.

In our study, we had a relatively small number of pediatric liver transplant recipients. The ROC analyses and the corresponding estimates of sensitivity and specificity pooled 212 longitudinal sample episodes obtained from 40 patients. Because repeated observations from the same patient are correlated, this episode-level approach is subject to within-patient autocorrelation and may produce overly precise confidence intervals or optimistic estimates of diagnostic performance. Therefore, the GcfDNA cutoff of 10.21% and tacrolimus trough concentration cutoff of 7.75 ng/mL should be regarded as exploratory rather than clinically validated thresholds. Before clinical implementation, both cutoffs require validation in independent external cohorts using patient-level validation and statistical methods that account for repeated measurements and relevant covariates, such as mixed-effects models or generalized estimating equations. In addition, because patients with active infections and biliary complications were excluded, the 10.21% GcfDNA threshold was optimized specifically to differentiate acute rejection from otherwise stable post-transplant courses. It should not be interpreted as a universal cutoff for distinguishing rejection from all causes of graft injury. In routine clinical practice, infectious and biliary complications are frequent and can independently elevate GcfDNA; the specificity of this threshold is therefore expected to be lower in an unselected population, necessitating a more comprehensive differential diagnostic workup. Also, because we focused exclusively on acute rejection within the first month after LDLT, our findings cannot be generalized to later post-transplant periods. This study was conducted exclusively in the Chinese pediatric liver transplant population, and we do not know whether our findings pertain to other ethnic groups.

For the present analysis, we enrolled a rigorously screened cohort after excluding patients who had nonrejection-associated allograft lesions. Although this stringent selection enabled specific identification of acute rejection-derived alterations, it inevitably limits the generalizability and real-world diagnostic specificity of the predefined cutoff value. Future prospective investigations should include the following actions: (1) externally validating the 10.21% GcfDNA cutoff in multicenter pediatric liver transplant cohorts; (2) including real-world competing diagnoses such as biliary complications, cytomegalovirus or Epstein-Barr virus infection, vascular complications, abdominal infection, and drug-induced liver injury; (3) integrating GcfDNA with miRNA, C-X-C motif chemokine ligand 10 (CXCL10, also termed IP-10), donor-specific antibodies, LFT trajectories, and imaging findings; (4) using methylation- or fragmentomics-based cfDNA methods to identify tissue or cell type of origin; and (5) pharmacogenetic studies incorporating donor and recipient CYP3A5/CYP3A4 genotypes to refine tacrolimus dosing.

In this single-center exploratory study, GcfDNA demonstrated potential diagnostic value for detecting graft injury Specifically, after the early postoperative phase marked by pronounced analytical variability, a GcfDNA cutoff value of 10.21% may serve as a novel and exploratory biomarker for monitoring acute rejection in pediatric patients following liver transplantation, although further validation is warranted. Furthermore, our findings indicate that a tacrolimus trough concentration of 7.75 ng/mL could represent the minimally effective level in children during the first month post-LDLT. Given the established safety profile of tacrolimus, these literature-supported findings endorse a therapeutic strategy targeting trough concentrations below 10 ng/mL to balance efficacy against the risk of drug-related toxicity, although this approach warrants confirmation in larger multicenter cohorts prior to widespread clinical implementation.

## Data Availability

De-identified individual-level data supporting the findings of this study have been deposited in the Zenodo repository (DOI: 10.5281/zenodo.22027130) and are publicly available.

## Supplementary material

SUPPLEMENTARY TABLE 1 Detailed characteristics of biopsyproven acute rejection episodes (n=20).

SUPPLEMENTARY FIGURE 1 Flow diagram of study screening and participant exclusion.

SUPPLEMENTARY FIGURE 2 Individual longitudinal trajectories of GcfDNA (%) from POD 1 to POD 28 in the 20 recipients with biopsy-proven acute rejection. Vertical arrows indicate the exact POD at which histological diagnosis of acute rejection was established for each patient. Abbreviations: GcfDNA, graft-derived cell-free DNA; POD, postoperative day.

SUPPLEMENTARY FIGURE 3 Distribution of CYP3A5 genotypes in recipients and donors.

SUPPLEMENTARY FIGURE 4 Graphical Abstract. Abbreviations: C0, trough concentrations; GcfDNA, graft-derived cell-free DNA; POD, postoperative day.
