# Supplementary material for: Dynamic kinetics of graft-derived cell-free DNA and personalized tacrolimus target ranges for acute rejection monitoring in pediatric liver transplantation
Source: Front Pharmacol. 2026 Sep 30;17:1939581. doi: 10.3389/fphar.2026.1939581 (PMC13662168; doi:10.3389/fphar.2026.1939581)
Supplement: Supplementary file 1 [file Table1.docx]

Supplementary Table 1. Detailed characteristics of biopsy‑proven acute rejection episodes (n=20)

| **Patient ID** | **Date of Liver Biopsy** | **Banff RAI Score** | **Rejection Grade** | **GcfDNANearest to Biopsy (%)** | **Tacrolimus C_0_ Nearest to Biopsy (ng/mL)** |
| --- | --- | --- | --- | --- | --- |
| 01 | POD 16 | 3 | borderline | 11.6 | 6.1 |
| 02 | POD 18 | 7 | moderate | 71.3 | 5.2 |
| 03 | POD 17 | 3 | borderline | 22.6 | 2.4 |
| 04 | POD 28 | 6 | moderate | 65.0 | 8.7 |
| 05 | POD 19 | 5 | mild | 39.1 | 7.3 |
| 06 | POD 13 | 4 | mild | 37.6 | 6.0 |
| 07 | POD 25 | 3 | borderline | 18.1 | 8.3 |
| 08 | POD 13 | 4 | mild | 21.8 | 6.5 |
| 09 | POD 10 | 9 | severe | 89.4 | 1.5 |
| 10 | POD 14 | 8 | severe | 44.7 | 6.5 |
| 11 | POD 12 | 4 | mild | 22.1 | 6.2 |
| 12 | POD 8 | 8 | severe | 47.8 | 6.2 |
| 13 | POD 9 | 7 | moderate | 48.4 | 7.1 |
| 14 | POD 22 | 6 | moderate | 31.0 | 5.1 |
| 15 | POD 21 | 6 | moderate | 35.7 | 7.1 |
| 16 | POD 14 | 3 | borderline | 24.8 | 5.0 |
| 17 | POD 14 | 5 | mild | 31.9 | 6.1 |
| 18 | POD 14 | 9 | severe | 80.1 | 2.7 |
| 19 | POD 28 | 7 | moderate | 43.9 | 6.2 |
| 20 | POD 21 | 5 | mild | 38.6 | 5.8 |
